# Structure-based development of human antibody cocktails against SARS-CoV-2

**DOI:** 10.1038/s41422-020-00446-w

**Published:** 2020-12-01

**Authors:** Nan Wang, Yao Sun, Rui Feng, Yuxi Wang, Yan Guo, Li Zhang, Yong-Qiang Deng, Lei Wang, Zhen Cui, Lei Cao, Yan-Jun Zhang, Weimin Li, Feng-Cai Zhu, Cheng-Feng Qin, Xiangxi Wang

**Affiliations:** 1 CAS Key Laboratory of Infection and Immunity, National Laboratory of Macromolecules, Institute of Biophysics, Chinese Academy of Sciences, Beijing, 100101 China; 2 University of Chinese Academy of Sciences, Beijing, 100049 China; 3 Department of Respiratory and Critical Care Medicine, West China Medical School/West China Hospital, Sichuan University, Chengdu, Sichuan 610041 China; 4 State Key Laboratory of Pathogen and Biosecurity, Beijing Institute of Microbiology and Epidemiology, AMMS, Beijing, 100071 China; 5 National Health Commission of the People’s Republic of China, Key laboratory of Enteric Pathogenic Microbiology (Jiangsu Provincial Center for Disease Control and Prevention), Nanjing, Jiangsu 210009 China; 6 Department of Microbiology, Zhejiang Provincial Center for Disease Control and Prevention, Hangzhou, Zhejiang 310000 China; 7 Guangzhou Regenerative Medicine and Health Guangdong Laboratory, Guangzhou, Guangdong 510200 China

**Keywords:** Cryoelectron microscopy, Molecular biology

Dear Editor,

The ongoing COVID-19 pandemic caused by severe acute respiratory syndrome coronavirus 2 (SARS-CoV-2) has resulted in unprecedented public health and socioeconomic crises, requiring urgent developments of effective COVID-19 therapeutics and vaccines. Humoral immunity is essential for protection against coronavirus infections and passive immunization has been demonstrated to be effective in curing SARS-CoV-2-challenged nonhuman primates.^1,2^ A deep understanding of the molecular basis of neutralizing antibody (NAb) responses to SARS-CoV-2 could facilitate vaccine design and drug discovery. Spike (S) protein, the major protective antigen, utilizes its receptor-binding domain (RBD) to engage the host receptor ACE2 for viral entry into host cell. Subsequently, a number of RBD-targeting NAbs against SARS-CoV-2, which block the binding of S to ACE2 have been reported and characterized.^2–5^ However, a major concern is the emergence of numerous viral mutations within RBD, in particular, when selective pressure is applied in immunotherapies, resulting in resistance against these antibodies. Recently, two antibodies targeting N-terminal domain (NTD) of S exhibited potent neutralizing activities against SARS-CoV-2.^6,7^ When used in combination with RBD-targeting and NTD-directing NAbs, the protective effect was magnified.^5,6^ Thus, a combination of antibodies could not only increase the potency of protection, but also prevent viral escape of immune responses via mutations. These preliminary results highlight the benefits of using a cocktail of antibodies for treating COVID-19 and provide a framework for rational design of antibody cocktail therapeutics that target both RBD and NTD regions. Furthermore, the structural characterizations of S in complex with potential NAb cocktails reported recently inform strategies for the development of vaccines for protection against COVID-19.

Successful antibody cocktail therapeutics for Ebola were generated by mixing NAbs from humanized antibodies from mice and human survivors. This indicates that NAb diversity plays critical roles in the design of antibody cocktails, and that this diversity can be achieved through various approaches. We have recently described parallel efforts, involving use of humanized antibodies developed from libraries of mouse origin and antibodies screened from libraries constructed from peripheral blood mononuclear cells (PBMCs) of convalescent humans. A large collection of highly potent NAbs targeting both RBD and NTD of SARS-CoV-2 S protein were obtained.^3–6^ Among these, two humanized RBD-targeting NAbs, named H014 and HB27, are pan-SARS-CoVs cross-reactive and SARS-CoV-2-specific NAbs, respectively.^3,4^ The other two fully human NAbs, FC05 and P17, recognize NTD and RBD of S, respectively.^5,6^ All four NAbs individually exhibited potent neutralizing activities at sub-nM concentrations and conferred effective protection against SARS-CoV-2 in animal models.^3–6^ These preliminary results allowed us to rationally design two-antibody cocktails of the NTD-targeting FC05 in combination with the RBD-targeting NAbs. We firstly evaluated the simultaneous binding of FC05 and three RBD-directing NAbs to S by competitive surface plasmon resonance (SPR). The CM5 sensor labeled with SARS-CoV-2 S trimer was saturated with FC05 and flooded with H014 or HB27 or P17 in the flow through (Fig. 1a). As expected, the binding of FC05 does not affect the attachment of any of the three RBD-specific NAbs to the SARS-CoV-2 S trimer, underlining the potential of these antibodies in formulating cooperative two-antibody cocktails as they bind simultaneously to distinct domains (Fig. 1a). Although more recently, synergistic effects between pairs of non-competing RBD-targeting NAbs have been reported for SARS-CoV-2, such as a pair consisting of H014 and P17,^5^ combinations of FC05 and H014 or HB27 or P17 that bind to NTD and RBD, respectively, of S, provide opportunities to develop more optimal antibody cocktail therapeutics for COVID-19.

**Fig. 1:**
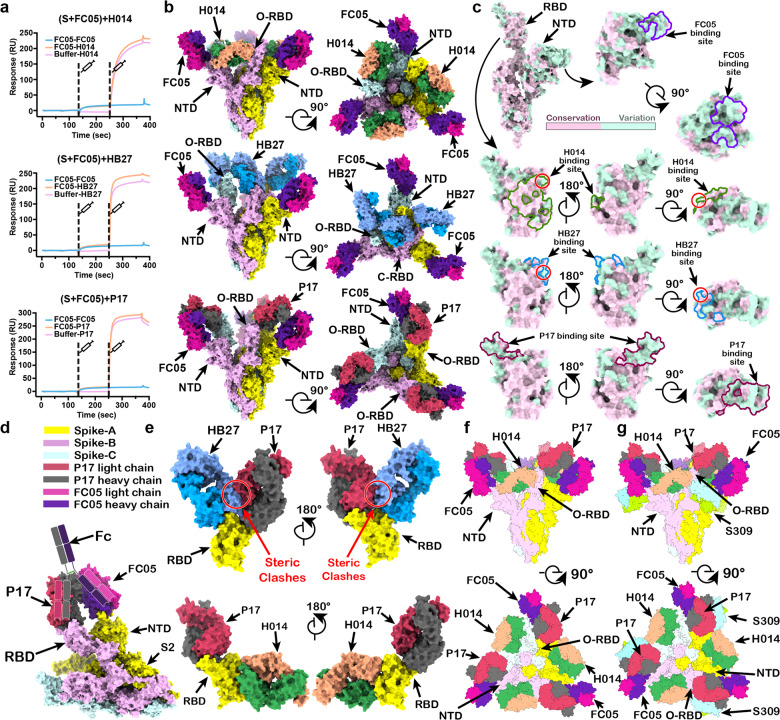
Structural basis for cooperativity in antibody cocktails. **a** SPR kinetics of competitive binding of two-antibody cocktails of FC05 and H014, FC05 and HB27 or FC05 and P17 to SARS-CoV-2 S. For all kinetics, S was immobilized onto sensor; FC05 was first injected, followed by H014 (upper), HB27 (middle) or P17 (lower). Control groups are depicted by blue curves. **b** Orthogonal views of FC05–H014–S (upper), FC05–HB27–S (middle), and FC05–P17–S (lower). Open-state RBD, and close-state RBD are labeled with O-RBD and C-RBD, respectively. **c** Surface representation of the S monomer or RBD. The areas buried by FC05, H014, HB27, and P17 epitopes are marked in purple, forest green, sky blue, and red lines, respectively. Sequence identities and differences between the S of SARS-CoV and SARS-CoV-2 are shown in pink and green, respectively, mapped on the surface of SARS-CoV-2 S/RBD. The overlapped residues of HB27 and H014 epitopes are marked with red cycles. **d** P17 and FC05 Fabs mimic the two “arms” of a single IgG molecule to bind S. A structure-based bivalent antibody is modeled. Fc, NTD, RBD, S2, P17 and FC05 are labeled. **e** Superimpositions of complex structures of S–P17 and S–HB27 as well as S–H014. Steric clashes between HB27 and P17 are highlighted with red cycles. **f**, **g** Orthogonal views of a three-antibody cocktail of FC05, H014, and P17 (**f**) and a four-antibody combination of FC05, H014, P17 and S309 (**g**).

A deep understanding of the molecular basis for synergistic neutralization by antibodies of cocktails and the identification of additional neutralizing epitopes for a hypothetical 3^rd^ or 4^th^ antibody partner to further reinforce the cocktail would aid the development of powerful rationally designed vaccine and therapeutics. Structural investigations of mechanisms underlying the cooperativity between the antibodies of cocktails during neutralization via targeting different domains, however, have not yet been carried out for SARS-CoV-2. In our previous studies, near-atomic structures of a prefusion stabilized SARS-CoV-2 S trimer in complex with individual Fab fragment (FC05 or H014 or HB27 or P17) have been determined by cryo-electron microscopy (cryo-EM) and epitopes for these four NAbs have been well characterized.^3–6^ Here, we further performed cryo-EM analysis of the SARS-CoV-2 S trimer in complex with three pairs of antibody cocktails (FC05 and H014; FC05 and HB27; FC05 and P17) with overall resolution of 3.4 Å–3.7 Å (Fig. 1b; Supplementary information, Figs. S1–S3 and Table S1). For each two-antibody cocktail, there are in total six copies of Fabs bound to one S trimer, where three FC05 Fabs bind on the side of each NTD and three RBD-directing Fabs bind at the side (H014) or top of each RBD (HB27 and P17), shielding most of the regions of S1 (Fig. 1b). Distinct from most structural studies of the apo SARS-CoV-2 S trimer in which multiple conformational states corresponding to 0–3 RBDs open were observed,^3–8^ only one conformational state was observed in the structures of S in complex with each two-antibody cocktail (Fig. 1b), which also differed from the structural insights gained from the SARS-CoV-2 S in complex with FC05 or H014 or P17 alone. Furthermore, each of the two-antibody cocktail limits the conformational transitions of RBD, suggesting an additional benefit conferred during the cooperative neutralization carried out by the cocktail antibodies via interfering with viral membrane fusion. Functional assays have previously shown that obstruction of conformational transitions of S protein by NAbs, can activate or inhibit fusion of the coronavirus with the host cell membrane.^4,5,9^

FC05 recognizes an extremely variable patch on the NTD; none of the bound residues are conserved between SARS-CoV and SARS-CoV-2, explaining the virus-specific binding and neutralization activities of FC05 (Fig. 1c). Contrarily, H014 targets a highly conserved epitope on the side of RBD and the epitope is only accessible when RBD is in the open state (Fig. 1c). Remarkably, FC05 and H014 adopt an interdigitated arrangement surrounding the exterior of the S trimer apex, which completely blocks the domain swapping between protomers and prevents the closure of RBD (Fig. 1b). It is worthy to note that the cocktail of FC05 and H014 represents a combination of two NAbs generated from two different approaches which confer potent neutralization activities against pan-SARS-CoVs. Interestingly, three HB27 Fabs bind at the top of each RBD, either in the open or closed state, forming a “cap” layer at the trimer apex close to the pseudo-threefold axis. This “cap” layer together with an exterior layer formed by three FC05 molecules, fully occlude the receptor binding site. The antibodies also prevent access to the proteolysis sites, cutting off the proteolytic activation of S by cell surface proteases (Fig. 1b, c). Surprisingly, P17 and FC05 exhibit a parallel binding mode to RBD and the adjacent NTD, forming a “two-Fab bundle” structure with contacts at the constant domains of these two Fabs (Fig. 1b–d). Structural characterization revealed that P17 and FC05 Fabs can mimic the two “arms” of a single IgG molecule, allowing us to rationally design a divalent antibody against SARS-CoV-2 (Fig. 1d). Footprint analysis of epitopes of these four NAbs suggests a partial overlap between H014 and HB27 epitopes, but no overlap between H014 and P17 or between HB27 and P17 epitopes (Fig. 1c). Superimposition of the structures of the complexes of S–HB27 and S–P17 shows steric clashes between HB27 and P17, suggesting that these two antibodies may not be able to bind the same RBD simultaneously (Fig. 1e). Theoretically it is plausible to develop a three-antibody cocktail consisting of FC05, H014 and P17 that simultaneously target three distinct regions^5^ (Fig. 1e, f). To further investigate whether a fourth partner exists, available structures of NAbs against SARS-CoV-2 were aligned to our complex structures. Interestingly, S309, another cross-reactive NAb^10^ against SARS-CoV-2 and SARS-CoV has a potential to constitute the fourth component for our cocktails (Fig. 1g), conferring synergy in protective efficacy against pan-SARS-CoVs as well as robustness to viral mutation escape.

In summary, we applied a rational NAb screening strategy to two different approaches of constructing antibody libraries, yielding potent SARS-CoV-2 neutralizing antibodies with high diversity. SPR-based cross-competition assays and cryo-EM analysis guided the development of next-generation human NAb cocktail, which can confer broad and effective protection against pan-SARS-CoVs.

## Supplementary information

Supplementary Information

## Data Availability

Cryo-EM density maps of the FC05–H014–S, FC05–HB27–S and FC05–P17–S complexes have been deposited at the Electron Microscopy Data Bank with accession codes EMD-30486, EMD-30487 and EMD-30488, and related atomic models have been deposited in the protein data bank under accession codes 7CWS, 7CWT and 7CWU, respectively.

## References

[1] Gao Q (2020). Science.

[2] Shi R (2020). Nature.

[3] Lv Z (2020). Science.

[4] Zhu, L. et al. *bioRxiv*10.1101/2020.11.24.393629 (2020).

[5] Yao, H. et al. *Cell Res.* 10.1038/s41422-020-00444-y (2020).

[6] Zhang, L. et al. *bioRxiv*10.1101/2020.09.23.309294 (2020).

[7] Chi, X. et al. *Science ***369,** 650–655 (2020). 10.1126/science.abc6952 PMC7319273 32571838

[8] Gui M (2017). Cell Res..

[9] Walls AC (2019). Cell.

[10] Pinto D (2020). Nature.

