# Supplementary material for: Structure-based development of human antibody cocktails against SARS-CoV-2
Source: Cell Res. 2020 Dec 1;31(1):101–3. doi: 10.1038/s41422-020-00446-w (PMC7705432; doi:10.1038/s41422-020-00446-w)
Supplement: Supplementary file 1 — Supplementary Information [file 41422_2020_446_MOESM1_ESM.pdf]

Supplementary information

Methods and Materials

SARS-CoV-2 S trimer cloning, expression and purification

The sequence for expressing SARS-CoV-2 S trimer (residues 1-1208, GenBank: MN908947.3) was inserted into the vector pCAGGS with a T4 fibritin trimerization motif, an HRV3C protease cleavage site and a twin-Strep-tag in the C terminus. The gene of S protein was constructed with two proline substitutions at residues 986 and 987 as well as a "GSAS" instead of "RRAR" at the furin cleavage sites as previously reported[11]. Then the expression vector was used to transiently transfect HEK Expi 293F cells (Thermo Fisher Scientific) by polyethylenimine. SARS-CoV-2 S trimer was purified from the filtered cell supernatants using StrepTactin resin (IBA). For further purification, the eluted sample was concentrated and subjected to additional purification by size-exclusion chromatography in 20 mM Tris, 200 mM NaCl, pH 8.0.

Cryo-EM sample preparation, data acquisition and structure determination

All the purified Fab fragments and purified SARS-CoV-2 trimer were diluted to 1 mg/ml. FC05 Fab fragments were firstly mixed with Fab fragments of H014, HB27 or P17, respectively. Then each mixture was incubated with purified SARS-CoV-2 trimer at a mol ratio of 3:1 at ice for 3 min. The C-flat 1.2/1.3 Au grid was glow-discharged and transferred with a 3 μl incubated aliquot. After 3 s blotting in 100% relative humidity, the grid was plunged into liquid ethane in Vitrobot (Thermo Fisher Scientific). All three Cryo-EM datasets were collected at 300 kV using Titan Krios microscope (Thermo Fisher Scientific) equipped with a K2 detector (Gatan, Pleasanton, CA). Movies were recorded at 32 frames, with total dose of 60 $e^{-}Å^{-2}$ and a 1.5-2.7 μm defocus using serialEM yielding the final pixel size of 1.04 Å.

Totally 1,398 stacks for FC05-P17-S complex, 1,021 stacks for FC05-H014-S complex, and 1,532 stacks for FC05-HB27-S complex were recorded. RelionCorr[12] was used to correct beam induced motion and average frames. The GPU accelerated Gctf[13] was used to estimate CTF parameters. Micrographs with bad CTF parameters and ice were removed manually. The left micrographs were used to pick particles with Relion AutoPick. Totally 153,221, 194,304, and 186,929 particles for FC05-P17-S, FC05-H014-S, and FC05-HB27-S were picked out and used for 2D and 3D classification to remove garbage particles, yielding a total of 97,455, 99,382, and 110,356 particles for the final reconstruction. After the high-resolution refinement and

postprocess in Relion[13], three reconstructions with the resolution of 3.5 Å for FC05-P17-S, 3.4 Å for FC05-H014-S, and 3.7 Å for FC05-HB27-S were obtained. All the resolutions were evaluated on the basis of the gold-standard Fourier shell correlation (threshold = 0.143). The local resolution was evaluated by ResMap[14].

Structure of SARS-CoV-2 S in complex with FC05 and H014 or HB27 or P17 (Protein Data Bank ID:7D4G, 7CAH, 7CYP and 7CWO) were manually fitted into the refined EM maps using Chimera[15], corrected manually with COOT[16], and refined with phenix_real_space_refine[17]. The final models were evaluated by Molprobity[18] and all the statistics of datasets and refinement are summarized in Table S1.

Surface plasmon resonance

SARS-CoV-2 S trimer was immobilized onto a CM5 sensor chip surface using the NHS/EDC method to a level of ~1,800 response units (RUs) using Bia-core T100 (GE Healthcare) and a PBS running buffer (suspended with 0.05% Tween-20). Purified FC05, H014, HB27 and P17 were prepared for the competitive binding assays. The first sample FC05 flew over the chip at a rate of 20 ul/min for 120s, then the other three different antibodies individually were injected at the same rate for another 120s. The FC05 antibody was used as a negative control. All antibodies were evaluated at saturation concentration of 500 nM, except for FC05 (1000 nM). All antibodies were regenerated with 35 mM NaOH. The response of the three antibodies to the FC05 was recorded at room temperature and the data was analyzed using Bia-core T100 Evaluation Software (GE Healthcare).

Supplementary Figures

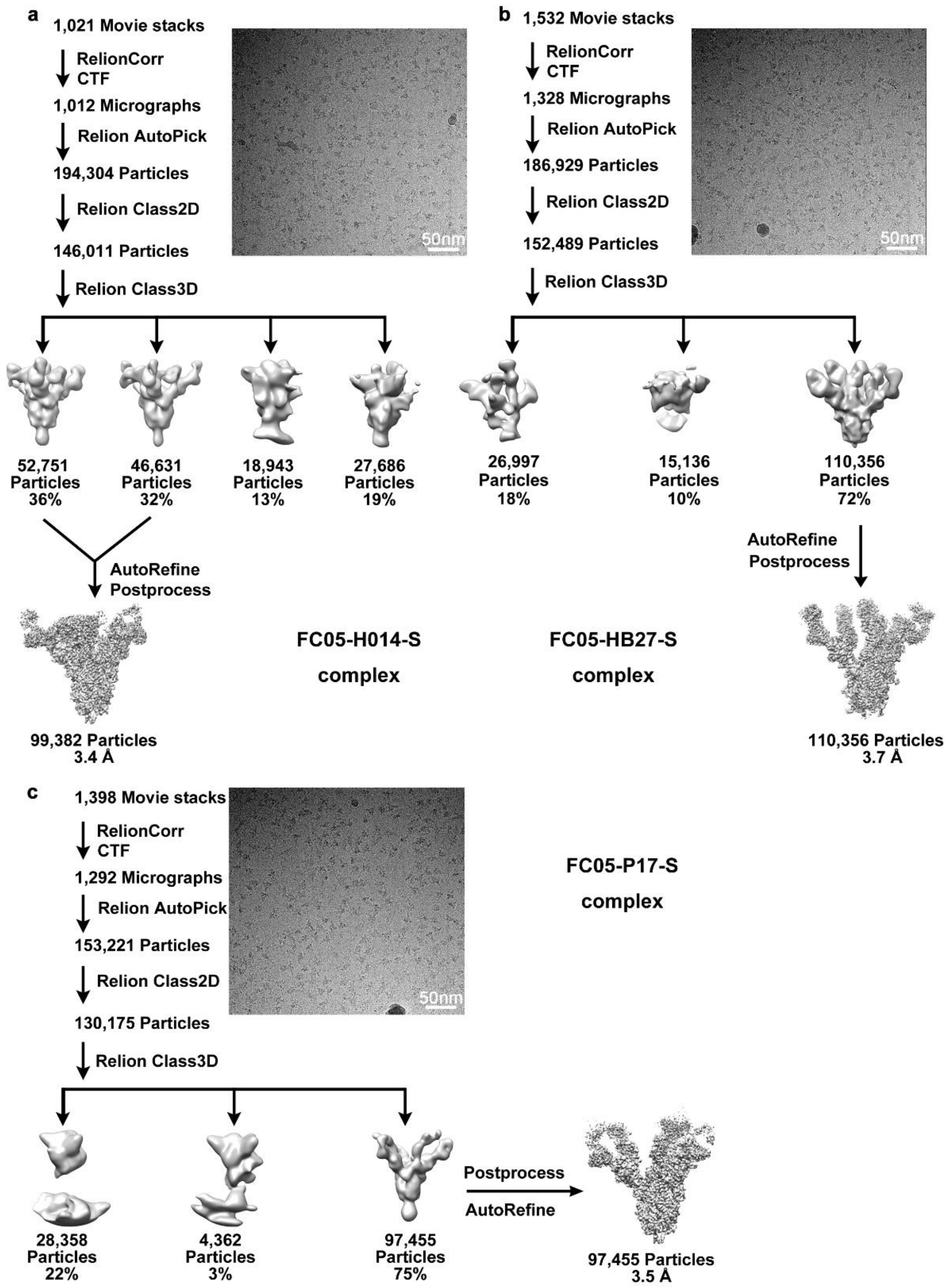

Supplementary information, Fig. S1 Cryo-EM images and Data processing flowcharts. (a) FC05-H014-S complex; (b) FC05-HB27-S complex; (c) FC05-P17-S complex.

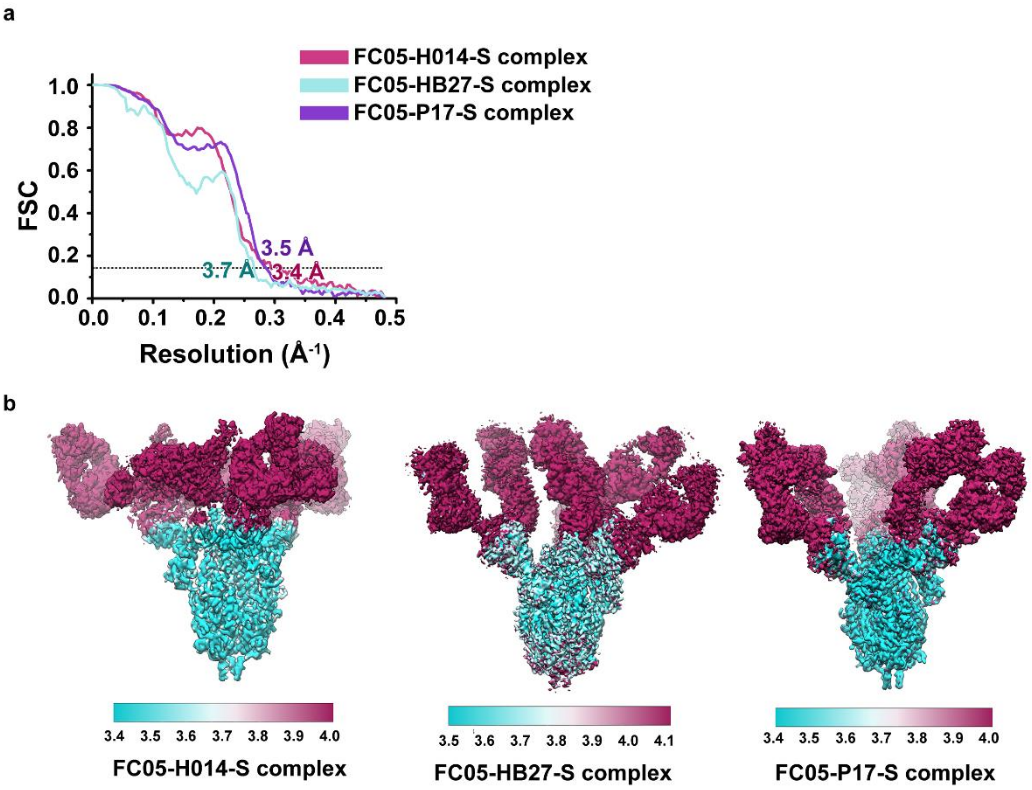

Supplementary information, Fig. S2 Cryo-EM images and resolution evaluation of the EM maps of FC05-H014-S, FC05-HB27-S and FC05-P17-S complexes. (a) The gold-standard FSC curves of the final maps. (b) Local resolution assessments of cryo-EM maps. Local-resolution evaluation of the maps of FC05-H014-S, FC05-HB27-S, and FC05-P17-S complexes using ResMap[14] are shown.

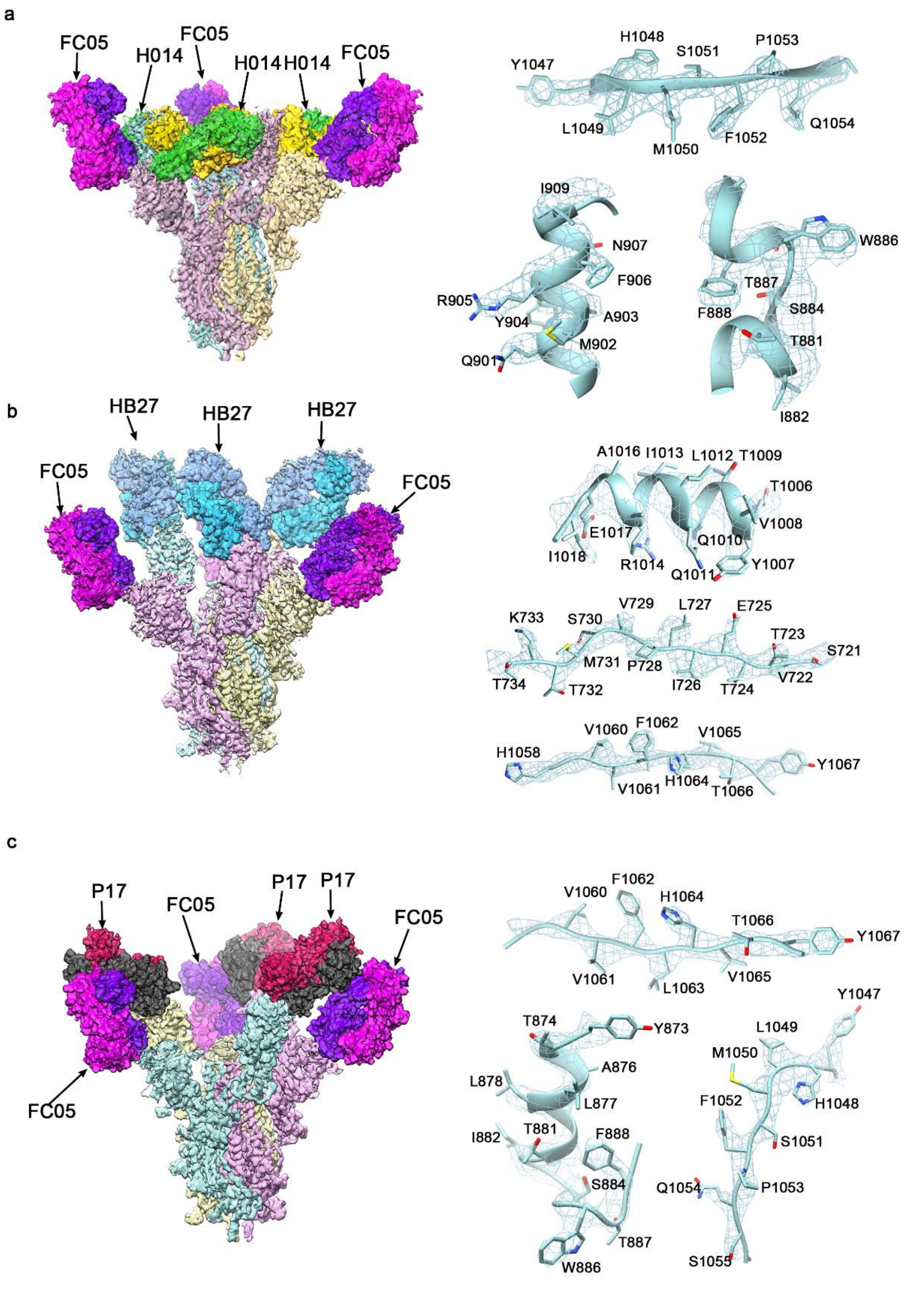

Supplementary information, Fig. S3 Cryo-EM density maps and atomic models. Cryo-EM maps of FC05-H014-S (a), FC05-HB27-S (b) and FC05-P17-S complexes (c) are shown. The right panels present the density maps (mesh) and related atomic models shown as sticks.

Supplementary information, Table S1 | Cryo-EM data collection and refinement statistics

Protein	FC05-H014-S (7CWS, EMD-30486)	FC05-P17-S (7CWU, EMD-30488)	FC05-HB27-S (7CWT, EMD-30487)
Magnification	81,000	81,000	81,000
Voltage (kV)	300	300	300
Electron dose ($e^-/Å^2$)	60	60	60
Defocus range (μm)	1.5-2.7	1.5-2.7	1.5-2.7
Pixel size (Å)	1.04	1.04	1.04
Symmetry imposed	C3	C3	C1
Detector	K2	K2	K2
Initial particle images (no.)	194,304	153,221	186,929
Final particle images (no.)	99,382	97,455	110,356
Map resolution (Å)	3.4	3.5	3.7
FSC threshold	0.143	0.143	0.143
Map resolution range (Å)	416-3.4	416-3.5	416-3.7
**Refinement**			
Initial model used (PDB code)	6VSB	6VSB	6VSB
Model resolution (Å)	3.46	3.46	3.46
FSC threshold	0.143	0.143	0.143
Model resolution range (Å)	6.5-3.2	4.1-3.2	8.3-3.7
Map sharpening B factor ($Å^2$)	-51.2	-55.3	-85.2
Model composition			
Non-hydrogen atom	36,501	36,156	36,034
Protein residues	4,602	4,582	4,584
Ligands	54	54	42
B factors ($Å^2$)			
Protein	48.49	64.01	49.30
Ligand	25.67	27.56	30.00
R.m.s.d			
Bond lengths (Å)	0.011	0.008	0.011
Bond angles (°)	1.28	1.24	1.44
Validation			
MolProbity score	2.53	2.32	2.58
Clashscore	35	21	32
Poor rotamer (%)	0.18	0.2	1.59
Ramachandran statistics			
Favored (%)	91.68	91.70	93.37
Allowed (%)	8.06	8.08	6.01
Outliers (%)	0.27	0.22	0.62

Supplementary references

11 Wrapp, D. *et al.* Cryo-EM structure of the 2019-nCoV spike in the prefusion conformation. *Science* **367**, 1260-1263 (2020).

12 Scheres, S. H. Processing of Structurally Heterogeneous Cryo-EM Data in RELION. *Methods in enzymology* **579**, 125-157 (2016).

13 Zhang, K. Gctf: Real-time CTF determination and correction. *Journal of structural biology* **193**, 1-12 (2016).

14 Kucukelbir, A., Sigworth, F. J. & Tagare, H. D. Quantifying the local resolution of cryo-EM density maps. *Nature methods* **11**, 63-65 (2014).

15 Yang, Z. *et al.* UCSF Chimera, MODELLER, and IMP: an integrated modeling system. *Journal of structural biology* **179**, 269-278 (2012).

16 Emsley, P. & Cowtan, K. Coot: model-building tools for molecular graphics. *Acta Crystallographica Section D: Biological Crystallography* **60**, 2126-2132 (2004).

17 Afonine, P. V. *et al.* Towards automated crystallographic structure refinement with phenix. refine. *Acta Crystallographica Section D: Biological Crystallography* **68**, 352-367 (2012).

18 Chen, V. B. *et al.* MolProbity: all-atom structure validation for macromolecular crystallography. *Acta Crystallographica Section D: Biological Crystallography* **66**, 12-21 (2009).
